# Where and Why Modeling Amyotrophic Lateral Sclerosis

**DOI:** 10.3390/ijms22083977

**Published:** 2021-04-12

**Authors:** Francesco Liguori, Susanna Amadio, Cinzia Volonté

**Affiliations:** 1 Preclinical Neuroscience, IRCCS Santa Lucia Foundation, 00143 Rome, Italy; f.liguori@hsantalucia.it (F.L.); s.amadio@hsantalucia.it (S.A.); 2 Institute for Systems Analysis and Computer Science “A. Ruberti”, National Research Council (IASI—CNR), 00185 Rome, Italy

**Keywords:** amyotrophic lateral sclerosis, animal modeling, *Saccharomyces cerevisiae*, *Caenorhabditis elegans*, *Drosophila melanogaster*, *Danio rerio*, rodents, canines, non-human primates

## Abstract

Over the years, researchers have leveraged a host of different in vivo models in order to dissect amyotrophic lateral sclerosis (ALS), a neurodegenerative/neuroinflammatory disease that is heterogeneous in its clinical presentation and is multigenic, multifactorial and non-cell autonomous. These models include both vertebrates and invertebrates such as yeast, worms, flies, zebrafish, mice, rats, guinea pigs, dogs and, more recently, non-human primates. Despite their obvious differences and peculiarities, only the concurrent and comparative analysis of these various systems will allow the untangling of the causes and mechanisms of ALS for finally obtaining new efficacious therapeutics. However, harnessing these powerful organisms poses numerous challenges. In this context, we present here an updated and comprehensive review of how eukaryotic unicellular and multicellular organisms that reproduce a few of the main clinical features of the disease have helped in ALS research to dissect the pathological pathways of the disease insurgence and progression. We describe common features as well as discrepancies among these models, highlighting new insights and emerging roles for experimental organisms in ALS.

## 1. Introduction

Amyotrophic lateral sclerosis (ALS) was identified more than two hundred years ago and incredible scientific progress has been made during the past decades although there is still a shortage of information and many hypotheses have still to be confirmed about its complex pathogenesis [1,2]. Very recently, a high-throughput-omics analysis has contributed to the identification of candidate causal and risk ALS genes, molecular alterations and derangements of pathways along with their impact on the outcome of the disease [3,4]. Most research has been carried out and achievements also obtained thanks to the exploitation of several different in vitro and in vivo experimental models. These provide essential clues for: (a) understanding the genesis of motor neuron death as a hallmark of the disease; (b) distinguishing the overall contribution of genes, protein targets and mechanisms involved in the insurgence and progression of ALS; (c) validating new biomarkers for diagnostic purposes; (d) discovering and repurposing new treatments comprising drugs, antibodies, stem cells and gene therapies [5,6,7,8,9]. However, the use of a single model cannot per se discern and redirect the pathological process of the disease. Only the integrated and comparative scrutiny of different ALS organisms will be conceivably able to provide the right answers to the several still open questions about ALS. The present work will provide an updated review of the experimental ALS models generated in both invertebrate and vertebrate organisms that have helped to promote fruitful research in the ALS field.

## 2. ALS: An Old But Unbeaten Disease

### 2.1. ALS Genes

A major part of ALS cases is classified as “sporadic” (sALS) while up to 10% are defined as “familial” (fALS) in origin. Variations in a number of genes and loci are associated with ALS susceptibility. More than 50 genes have been associated with fALS [10] and in the present review we will focus on the most commonly mutated fALS genes (*SOD1*, *FUS*, *TDP-43* and *C9ORF72*), which are, overall, involved in more than half of fALS cases and for which many models have been developed so far.

*SOD1* was the first gene to be recognized as ALS-linked [11,12]; it encodes for a ubiquitous Cu/Zn superoxide dismutase that catalyzes the dismutation of superoxide radicals to hydrogen peroxide and dioxygen. Over the years, more than 150 mutations of the *SOD1* gene have been related to ALS [13] but the mechanism by which mutant *SOD1* triggers motor neuron damage remains still unclear. To date, overwhelming evidence supports the combination of both loss- and gain-of-toxic-functions linked to *SOD1* mutations [14,15,16] and leading to the perturbation of mitochondrial dynamics, protein folding, axonal transport and cellular metabolism [17,18]. While mutant SOD1 is recognized as a pathological cause of about 20–25% of fALS cases, the wild type or misfolded SOD1 is implicated also in a significant fraction of sALS cases [19]. Mutant SOD1 protein expression in non-neuronal cells is also involved in ALS pathogenesis [20,21].

Another ALS-related gene is *FUS*, coding for the multirole protein Fused-in-Sarcoma/Translocated in Liposarcoma (FUS/TLS) present in RNA-containing stress granules [22,23,24] and involved in RNA metabolism [25,26,27] and gene expression [28]. The protein belongs to the FET (FUS-EWS-TAF15) RNA-binding protein family and the gene was recently described as bicistronic, encoding also for an additional peptide (altFUS) [29]. Since 2009, several mutations in the *FUS* gene have been linked to ALS (with a frequency of 2.8% for fALS cases and 0.3% for sALS cases) [30,31,32,33,34,35], many of which affect FUS nuclear localization, impairing its role as a regulator of transcription and RNA maturation and causing toxic FUS aggregates in the cytoplasm [36,37,38,39,40,41]. Mutant FUS also confers loss- and gain-of-functions in the nucleus by inducing the abnormal formation of dysfunctional subnuclear bodies known as paraspeckles of which FUS is an essential component [42].

Found as the primary component of neuronal inclusions in ALS and fronto-temporal dementia (FTD) patients [43,44], TAR-binding protein 43 (TDP-43) is involved in several functions relating to RNA metabolism such as mRNA stability [45], miRNA processing [25] and splicing regulation [46]. More than 50 different point mutations in the *TARDBP* gene (encoding for TDP-43) have been identified as ALS-causing (frequency: 4.2% in fALS; 0.8% in sALS) [34,35], altering its shuttle role from the nucleus to the cytoplasm and therefore modulating its RNA-related functions and determining the formation of aggregates [47]. In spinal motor neurons, TDP-43 binds to low molecular weight neurofilament mRNA, thus suggesting roles also in regulating axonal stability or damage. Moreover, TDP-43 participates in the non-homologous end joining enzymatic pathway and repair of double-strand DNA breaks in stem cell-derived motor neurons while its absence is correlated with increased DNA breaks [48]. Finally, a hyperphosphorylated, ubiquitinated and cleaved form of TDP-43 is considered to be a disease-relevant protein in ALS [49].

The *Chromosome 9 Open Reading Frame 72 (C9ORF72)* gene contains 12 exons and encodes for a little characterized protein that has a pivotal role in autophagy modulation, recently described as part of a guanine nucleotide exchange factor complex [50,51]. In a healthy condition, the hexanucleotide GGGGCC (G_4_C_2_) within the first intron may be repeated from 2 to 23 times but its aberrant expansion reaching hundreds or thousands of repeats has been found in ALS and FTD patients and is considered the most common genetic cause of fALS. It is also detected in occasional patients with sALS with a frequency of 39% and 7%, respectively [52,53,54,55,56]. Although still debated, mounting evidence suggests that *C9ORF72*-ALS pathogenesis is the result of a cascade of multiple cellular mechanisms that include: (i) RNA toxicity depending on G_4_C_2_ hexanucleotide repeat expansions (HRE) [57,58]; (ii) aggregation of toxic dipeptide repeat proteins (DPR) produced from the HRE region through Repeat-Associated Non-ATG (RAN) translation [59,60,61]; (iii) reduced levels of C9ORF72 protein causing disease by loss-of-function mechanisms [62]. Although not mutually exclusive, all of these mechanisms may account for the disease-causing effects of the hexanucleotide *C9ORF72* expansions.

### 2.2. ALS Diagnostic Criteria

ALS is defined as a specific subtype of progressively degenerative motor neuron diseases that comprise primary lateral sclerosis, progressive muscular atrophy and progressive bulbar palsy and that are more generally classified as anterior horn cell diseases. These also include spinal muscular atrophy types I, II and III in children, type IV in adults and Kennedy’s disease, one of the most frequent disorders to be misdiagnosed as ALS [63,64]. However, ALS is not a disease that only harms motor neurons and several patients are currently reported to display extra motor deficits such as cognitive-behavioral disturbances, sensory nerve conduction failures and extrapyramidal alterations [65], thus questioning the classification of ALS as a mere motor neuron disease subtype. 

For several years, the only accepted criterion for diagnosing ALS was the electrodiagnostic examination, the chief laboratory methodology for testing the neuromuscular system, originally formulated by Lambert [66]. This diagnostic approach was then improved, included and revised in the newest ALS diagnostic tool for correct ALS diagnosis, i.e., the revised El Escorial Criteria [67,68]. 

Based on these criteria, ALS abnormalities must be present in multiple muscles with peripheral innervation from different nerve roots and in multiple limbs. In the revised El Escorial Criteria, the electrodiagnostic analysis is corroborated by accurate anamnesis and physical examination also via neuroimaging and laboratory testing, aimed at excluding other diseases that might mimic a generalized pathology of motor neurons. For instance, a reformulation of the electromyography and new diagnostic methods including transcranial magnetic stimulation, magnetic resonance imaging voxel-based morphometry and diffuse tensor imaging have also been introduced with the aim of further improving the diagnosis of ALS [69]. More recently, novel neuroradiological evidence sustained a procedure not only for diagnosing but also, most importantly, for staging sALS based on transactivation response TDP-43 pathology. According to this evidence, a corticofugal axonal model for the progression of ALS can be hypothesized whereby the pathology originates in the primary motor cortex and spreads via axonal projections to subcortical structures and to lower motor neurons [70]. Parallel experimental studies have also indicated the cell-to-cell propagation of aggregated or truncated TDP-43 protein suggesting a direct transmission of TDP-43 inclusions to contiguous cells [71]. An additional hypothesis in line with the anatomical spreading of TDP-43 aggregates now suggests a trans-synaptic transmission of TDP-43 [72]. In any case, the perturbation of cellular clearance seems to be accepted as a likely factor for modifying TDP-43 aggregation and disease spreading.

Despite this increasingly accumulated evidence [73], an accurate and early diagnosis of ALS is still impossible without reliable biomarkers. Although the explanation of some genetic and molecular bases of ALS have produced further advancement, early diagnostic detection remains challenging.

## 3. Modeling ALS in Different Systems

### 3.1. ALS In Vitro Models

An experimental tool that has greatly helped in understanding cellular and molecular mechanisms of ALS is represented by different neuronal cell lines expressing ALS genes. Since the first use of NSC-34 cells (a mouse neural hybrid cell line produced by a fusion of motor neuron-enriched embryonic mouse spinal cord neurons with mouse neuroblastoma cells [74]) stably transfected with a vector expressing a human wild type or G93A mutant *SOD1* [75,76,77,78,79], several works have continued to prove the resemblance of NSC-34 with motor neurons and their reliability as a cellular model of ALS. Similarly, the human neuroblastoma SH-SY5Y cell line has improved our understanding of not only SOD1 but also TDP-43-ALS pathology [80,81,82]. 

A great improvement in ALS cellular research was then provided by the exploitation of human-induced pluripotent stem cells (iPSCs) [83] that introduced the advantage of generating cells directly from individual patients with familial or sporadic ALS mutations [84,85]. In addition to motor neurons, iPSCs can also mimic other cell types associated with ALS such as astrocytes, microglia and oligodendrocytes, well known to be directly involved in the disease. Although iPSC technology eliminates the species-specificity problem inherent with the use of animal models, it remains an in vitro system with several limitations; for instance, a lack of tissue connectivity and functional complexity.

### 3.2. Use of Unicellular Eukaryotes for Modeling ALS

Despite unicellular models not being able to recapitulate complex disease phenotypes for certain, the completely sequenced genome [86] and the presence of an ortholog for many human disease genes [87] render the eukaryote budding yeast *Saccharomyces cerevisiae* a good candidate for where to model different pathologies [88,89,90]. As such, this model organism has been used to set up pioneering experiments identifying several different ALS genetic modifiers. For instance, a yeast ALS model led to the discovery that ATAXIN-2 modulates TDP-43 toxicity. Moreover, in yeast, *Ataxin-2* carrying a mild expansion of a CAG trinucleotide was identified as an ALS disease gene [91]. Likewise, the first evidence that stress granule components act as TDP-43 modifiers [92] and that nucleocytoplasmic transport is linked to *C9ORF72*-ALS [93] was carried out through unbiased yeast genetic screening. 

Human SOD1 expression in yeast is reported to assume a gain-of-toxic-function due to the loss of protein stability also perturbing metabolic regulation and enhancing the senescence process [94]. Interestingly, the heteromeric interaction between the wild type and mutant SOD1-A4V exacerbates the toxic effect and impairs the antioxidant response in *SOD1*-deficient yeast cells [95]. Yeast *SOD1*-deficiency is fully complemented by the expression of the human wild type SOD1, given the high identity between the two isoforms [96]. 

FUS ectopic overexpression in yeast inhibits the ubiquitin-proteasome system [97] and determines the formation of toxic cytoplasmic aggregates that co-localize with P-bodies and stress granules [24,87,98,99]. Removing the RNA recognition motif of FUS does not act on aggregates production but rescues FUS-induced toxicity, thus highlighting that the interaction between FUS and RNA is essential for FUS toxic action [98]. Consistently, a recent genetic screening in yeast identified several human genes acting as FUS toxicity suppressors, many of which were reported as RNA-binding proteins [100].

When expressed in yeast at high levels, TDP-43 forms subcellular aggregates that inhibit cell growth and exert a cytotoxic effect [101,102]. Interestingly, a yeast genetic screening revealed that the depletion of the Dbr1 debranching enzyme, whose role is to eliminate intronic lariats during the splicing process, becomes a strong suppressor of TDP-43-induced toxicity. Non-removed intronic lariats act as traps for TDP-43, thus impeding its interference with cellular RNAs and RNA-binding proteins [103,104]. Moreover, a recent report showed that TDP-43-induced cell death is considerably reduced in yeast deleted for *CNC1* (*Cyclin C*), *DNM1* (*Dynamin-related*) or *YBH3* (*Bax inhibitor*) genes, all involved in the oxidative stress response pathway at different levels [105].

Although *Saccharomyces cerevisiae* cannot offer insights concerning many aspects of the ALS pathomechanism as it is a unicellular organism, studies on yeasts have uncovered several key features of cytotoxicity and proteinopathy. Clearly, to gain a more comprehensive view of ALS pathology, it is mandatory to utilize higher complexity invertebrate and vertebrate models.

### 3.3. ALS Disease in Caenorhabditis elegans

A model system that is increasingly accepted and exploited in ALS is the nematode *Caenorhabditis elegans*, which is among the most intensely studied organisms in modern experimental biology. The simple and compact nervous system formed by 302 neurons and equipped with multiple synapses is well studied and amenable to neural circuit identification and analysis [106,107]. As such, *Caenorhabditis elegans* has been used to identify many genes affecting the nervous system function, development and disease states, given the fact that about 30% of human genes have a functional ortholog in the *C. elegans* genome [107]. Moreover, several mechanisms underlying the processing of sensory information, the generation of specific motor outputs and locomotion circuits or the control of behavioral states can be easily analyzed in the nematode. As many cellular stress and survival pathways are conserved in worms, transgenic *Caenorhabditis elegans* has been extensively used to model ALS as well [108].

In particular, the ubiquitous expression of mutant SOD1 prevents the natural biological response to oxidative stress and determines protein aggregation [109] while mutant SOD1 expression throughout the worm’s entire nervous system results in locomotion defects and an impaired neuronal transmission [110]. Interestingly, the generation of single-copy/knock-in mutant *SOD1* models (A4V, H71Y, L84V, G93A and G85R) allowed for the dissection of the specific impact of different mutations on cholinergic versus glutamatergic motor neuron degeneration, showing that ALS pathogenesis is neuronal subtype-specific and characterized by both gain-of-toxic-function and loss-of-function [111]. 

Regarding *FUS*, Murakami and colleagues expressed several *FUS* mutations and two truncated FUS proteins throughout the worm’s nervous system. Interestingly, only the mutations causing aggregation produced aberrant motor phenotypes that could not be rescued by the expression of a wild type *FUS*, thus suggesting a gain-of-function mechanism caused by these mutations [112]. Electron microscopy and electrophysiology assays moreover showed that the transgenic expression of a wild type or mutant *FUS* in *Caenorhabditis elegans* caused a decreased transmission from motor neurons to muscles and impaired synaptic vesicle docking at neuromuscular junctions [113].

The first TDP-43 overexpression model in the nematode was developed by Ash and co-authors. The pan-neuronal expression of human TDP-43 or *Caenorhabditis elegans* tdp-1 generates worms with uncoordinated, slow movements and the fasciculation of the GABAergic motor neurons [114,115]. Moreover, targeting the expression of mutant TDP-43 in worm GABAergic motor neurons causes age-dependent progressive paralysis, GABAergic neurodegeneration and impairment of synapses [116]. Moreover, mutant exogenous TDP-43 induces oxidative stress and the abnormal expression of endogenous *tdp-1* with a decline in neuronal functions and lifespan; coherently, *tdp-1* deletion determines the rescue of these phenotypes [117]. Locomotor defects in TDP-43 mutant worms were also rescued by *α*-metil-*α*-phenylsuccinimide treatment as shown by a disease modifier screening [118]. Recently, it has been established that hyperphosphorylation of TDP-43 interferes with protein homeostasis, resulting in neurotoxicity. Among the kinases involved, cell division cycle kinase 7 directly participates in TDP-43 phosphorylation because its inhibition reduces the phosphorylation of TDP-43 in *Caenorhabditis elegans* [119].

The nematode ortholog of *C9ORF72* is named *alfa-1* (*ALS/FTD associated gene homolog*). The decreased expression of *alfa-1* or loss-of-function of the mutant *alfa-1* causes motility defects, neurodegeneration (specifically of motor neurons) and sensitivity to osmotic stress [120]. Although further characterization remains to be done, it is interesting that the loss of *alfa-1* is linked to defective neuronal integrity specifically of GABAergic motor neurons in worms. Transgenic worms expressing a (G_4_C_2_)_29_ construct showed paralysis and lethality [121].

Although *C. elegans* modeling has allowed the recapitulation of a few aspects of ALS-related neurodegeneration and toxicity, it should be clearly considered that the nematode is a quite simple organism, lacking defined tissues and organs including a brain and blood. However, large screening using *Caenorhabditis elegans* for investigating genetic interactions among disease genes and targeting specific aspects of neurodegeneration seems promising and likely to show relevance also in higher organisms.

### 3.4. ALS Research by the Drosophila melanogaster Model

For more than a century *Drosophila melanogaster* has represented a valuable choice of where to perform studies from genetic to physiological fields of biology. Together with a copious progeny production at each generation, the availability of specific transgenesis programs allowing the fine-tuned control of transgene expression [122,123,124,125] has rendered *Drosophila* a very good platform for ALS modeling and therapeutic compound screening. This adds to the fact that transgenic flies expressing disease genes are able to reliably reproduce a few ALS symptoms (locomotor disabilities, cellular inclusions, mitochondrial dysfunction and early death) and that disease progression can be monitored through different kinds of tests [126]. For all of these reasons, most of the mutated genes known to be involved in ALS have been modeled in flies to assess their potential contribution to pathological phenotypes [127,128].

Transgenic flies expressing the human *SOD1* gene carrying point mutations (G85R, A4V, G37R, G41D) exhibit motor neuron dysfunction, climbing impairment, focal SOD1 aggregates, damaged mitochondria and oxidative stress [129,130,131]. Interestingly, different antioxidant compounds have been reported to be neuroprotective in SOD1-ALS *Drosophila* models, ameliorating motor performances, extending lifespan and lowering SOD1 cytoplasmic inclusions [132,133,134,135,136]. 

Flies expressing mutant human or *Drosophila FUS* (*cabeza*) showed progressive neurodegeneration, damaged photoreceptors and neuronal complications [137,138,139,140]. Interestingly, all defects were rescued by introducing a wild type human *FUS* transgene in a mutant *cabeza* background [141]. Studies on *FUS* transgenic flies have demonstrated that human FUS-induced toxicity is reduced by inhibiting nuclear export, thus confirming the involvement of nucleocytoplasmic transport in ALS pathogenesis [142,143]. Of note, a recent ALS modifier screening in two different *Drosophila* models carrying either a mutant *FUS* or mutant *TARDBP* human transgenes identified a complex array of enhancers and suppressors, many of which were able to rescue the ALS phenotype in both pathogenic conditions [144]. The identification of disease modifiers effective in different ALS models is surely key evidence of shared cellular and molecular pathways involved in many forms of ALS regardless of the causative genes. For instance, the overexpression of *ter94*, the fly ortholog of fALS-related gene *valosin-containing protein* (*VCP*) [145], was reported to be protective in a *Drosophila* model of *cabeza*-knockdown [146]. Additional studies on *Drosophila* allowed the correlation of FUS-induced neurodegeneration with many other cellular processes such as transcription and translation regulation [147,148], piRNA biogenesis [149], stress granule assembly [22,23,24,150,151] and Hippo-signaling pathways [152,153], shedding further light on the complex pathogenesis of ALS.

The overexpression of mutant or a wild type human TDP-43 as well as the overexpression of the *TBPH* (*TARDBP* fly ortholog) gene [139,154,155,156,157,158,159,160] in *Drosophila* impacts on lifespan, motor abilities, axonal transport and eclosion from the pupal case. *TBPH* depletion also causes locomotor disturbances and reduces the lifespan [159,161]. Of note, different potential therapeutic approaches have been found through *Drosophila* TDP-43 transgenic models. In particular, the modulation of autophagy [162,163,164], mitophagy [165], mitochondrial dynamics [166], glucose and lipid metabolism [167,168,169] and stress granule dynamics [92,170,171,172,173] have been reported to exert a positive effect on fly motor defects and lifespan.

The ectopic expression of an expanded *GGGCC* hexanucleotide or toxic dipeptide repeats in fly tissues causes ommatidia disorganization, motor defects and neuromuscular junction anomalies [174,175,176,177,178]. Recent studies on these gain-of-function *Drosophila* models revealed that different cellular processes impact on *C9ORF72*-ALS pathogenesis such as transcription [177,178,179,180], translation [181], nucleocytoplasmic transport [174,182,183,184] and protein degradation [185]. Despite the many advantages presented by the *Drosophila* model, it is clear that the highest limitation of this organism is the anatomical difference between the human and the fly brain together with the impossibility of conducting behavioral assays for which vertebrate studies are surely more informative.

### 3.5. ALS Pathogenesis in Danio rerio

Among the common model systems of ALS, a newcomer in the field is *Danio rerio* (zebrafish) that is becoming a widespread organism for investigating the disease owning to a lot of benefits in comparison with other vertebrate models such as a short life cycle, very high fertility and, most of all, larval transparency. Furthermore, zebrafish have a nervous system and main neurotransmitters remarkably similar to humans. Beside the high similarity with the human genome, genes strictly implicated in neurodegenerative diseases are also highly conserved between humans and zebrafish. Another advantage of using zebrafish is that the genome is easily modifiable [186,187,188]. 

In particular, transgenic *Danio rerio* overexpressing mutant SOD1 exhibited the main features of ALS (the loss of motor neurons, muscle degeneration, damage to neuromuscular junctions and impaired motor performance) culminating in decreased endurance in a swim test and the reduction of survival [189]. Moreover, in zebrafish expressing mutant SOD1, motor impairment, protein misfolding, ER-Golgi transport dysfunction and cytoplasmic mislocalization of TDP-43 were rescued by the redox function of the protein disulfide isomerase, suggesting that redox regulation is critical in maintaining cellular homeostasis [190]. 

The knockdown of endogenous *fus* or the overexpression of some human defective *fus* alleles in zebrafish generates a pathologic motor phenotype that is rescued by co-expressing the wild type human *fus* but not ALS-related *fus* mutations. The wild type human FUS also rescues the *tardbp* knockdown phenotype, suggesting a common pathogenic pathway. However, the co-expression of mutant *sod1* and mutant *fus* aggravates motor function more than the single overexpression of mutant *sod1* [186,191]. 

The expression of mutant *tardbp* (the gene encoding TDP-43) in zebrafish embryos induces motor neuron axonopathy, which was rescued by co-expressing the wild type but not mutant human *TARDBP* genes [192]. Recently, Campanari and collaborators confirmed in zebrafish the “distal axonopathy” hypothesis of ALS, according to which the pathological alterations take place at the neuromuscular junction during the presymptomatic phases of the disease. Zebrafish TDP-43 knockdown indeed resulted in early deficiencies of motor functions and neuromuscular junction disassembly. Moreover, the authors established that a partially depleted TDP-43 affected acetylcholinesterase expression, thus identifying this enzyme as a limiting factor regulating the connection between muscle and motor neurons [193].

Knockdown of the *C9ORF72* zebrafish ortholog induces axonopathy of motor neurons and locomotor deficits that, of note, are rescued by overexpressing human *C9ORF72* mRNA transcripts [194,195]. Conversely, G_4_C_2_ HRE results in RNA foci and dipeptide repeat formation leading to a significant increase of apoptotic cells, toxicity and motor axon abnormalities [196,197,198].

As for the other model systems, there are of course a few limitations in the use of zebrafish in ALS. Among these are the lack of upper motor neurons, the prevalent use of the embryonic stage and the lack of zebrafish antibodies for investigative studies [187,188].

### 3.6. Modeling ALS Phenotypes in Mouse, Rat and Guinea Pig Models

As the mouse (*Mus musculus*) has a development and a genome similar to humans, it is the most used system to reproduce neurodegenerative diseases including ALS [199]. Although much less used than mice, rats (*Rattus norvegicus*) and guinea pigs (*Cavia porcellus*) also have physiological characteristics like humans and, in some ways, are better models than mice because of their relatively larger brain and body size and higher stress resistance to experimental manipulation.

After discovering several mutations in the gene encoding the SOD1 enzyme as major causes of ALS [11], the most studied animal model has been the SOD1-G93A mouse [200]. However, because additional mutations have been discovered through the years, other mouse models have been developed such as the SOD1-D83G, SOD1-D90A and SOD1-G37R mice and are the most studied [201]. A common feature of these genetic variants with different copper-binding abilities and causing different phenotypes is to mimic the main features of ALS and to recapitulate the human disease [6,202]. For instance, the SOD1-G93A mouse displays abundant cytoplasmic inclusions and aggregates in degenerating motor neurons of ventral horns of the spinal cord together with reactive gliosis. Furthermore, impaired neuromuscular junction, extensive inflammation in the spinal cord and disruption of endothelial barrier integrity have been observed [9,203,204,205,206,207,208,209]. These features are also typical of the human pathology [210]. A major disadvantage of the SOD1-G93A mouse model is the absence of motor neuron degeneration in the cerebral cortex, one of the main features of the human disease [211]. However, further studies have shown a progressive decrease in dendritic length and spine density in the pyramidal neurons of the motor cortex, functional and structural neuronal alterations associated with abnormal glutamate activity in the motor cortex of presymptomatic G93A mice [212,213]. Interestingly, the transgenic SOD1-D83G mouse model showed a degeneration of motor neurons not only in the spinal cord but also in the cerebral cortex and particularly during early life but without showing any sign of paralysis in adult life, which was different from the SOD1-G93A mouse model [214]. Although the SOD1-D90A mutation appears to be less toxic than others, homozygous mice develop a fatal motor neuron disease with a slower progression and bladder disturbances similar to those observed in human ALS patients are homozygous for the D90A mutation. These mice accumulate SOD1 aggregates, inclusions and vacuoles in the ventral horns of the spinal cord [215]. Finally, SOD1-G37R mice also develop a progressive motor neuron disease. In particular, low levels of mutant SOD1 accumulation affect only the lower motor neurons while higher levels determine a more severe phenotype and also hit other neuronal populations. The main pathologic alteration of SOD1-G37R mice is the presence of vacuoles originating from mitochondrial debris in axons and dendrites [216].

Recently, Magota and co-workers demonstrated in the SOD1-G93A rat model that blood-spinal cord barrier destruction can directly contribute to motor neuron degeneration. The authors observed that the intravenous infusion of healthy mesenchymal stem cells to these rats delayed disease progression, preserved barrier function, increased the expression of the neurotrophic factor Neurturin and preserved motor neurons [217]. 

*Fus* mutations can now also be exogenously expressed in both rats and mice to mimic ALS [218]. In the rat model, motor dysfunction and paralysis, neuronal loss, gliosis and protein aggregation are observed in the brain and spinal cord. In the mouse model, in addition to protein aggregation and the consequent neurodegeneration of motor neurons, inhibition of the protein transport between the Golgi complex and endoplasmic reticulum and damage to the neuromuscular junction have also been described [219,220]. Moreover, Ishigaki and co-workers reported that *Fus* knockdown in the mouse hippocampus leads to Tau hyperphosphorylation, neuronal death and reduced adult neurogenesis. Furthermore, these animals exhibit phenotypic behavior typical of FTD such as limitations in socialization, hyperactivity and disinhibition [221,222].

In transgenic TDP-43 mice, damage to cortical and spinal motor neurons, axonal degeneration, mitochondrial dysfunction, gliosis and loss of neuromuscular junctions have been observed [44,223,224]. Recently, transgenic TDP-43 mice with a pathological accumulation of cytoplasmic TDP-43 were used to study DNA damage, an additional pathological feature of ALS. It was shown that the lack of DNA repair activity by TDP-43 mislocalization caused DNA damage [225]. 

Mice with *C9ORF72* mutations exhibit movement defects and cognitive deficits in addition to neuronal death, gliosis, weight loss, neuromuscular junction disruption, myopathy and behavioral changes such as hyperactivity and anxiety [53,54,210,219,226,227,228,229,230]. On the contrary, mice knockouts for *C9ORF72* do not show neurodegeneration or motor alterations [231].

On the other hand, the loss of C9ORF72 protein in rats requires the synergistic action of excitotoxicity to cause neuronal injury and disease progression. The ablation of C9ORF72 protein in rats and treatment with glutamate analogue kainic acid indeed stimulates the release of excitatory neurotransmitters resulting in an increased susceptibility of motor neurons to excitotoxicity, motor neuron degeneration and motor deficits [232].

Guinea pigs were also initially used to model ALS by adopting a diet deficient in ascorbic acid, which caused muscle atrophy, motor neuron degeneration and demyelination of the pyramidal tract [233]. However, guinea pigs were mainly used in pioneering studies to highlight an autoimmune etiology for ALS. In particular, defects of motor neurons have been experimentally induced by immunizing guinea pigs with purified bovine spinal motor neurons or homogenates from the ventral horns of healthy bovine spinal cords [234]. The serum from either immunized guinea pigs or from ALS patients was next used to infect mice through intraperitoneal inoculations. These mice demonstrated an increased acetylcholine release from axon terminals [235], IgG accumulation, increased intracellular calcium and morphological and functional defects of spinal motor neurons in part resembling ALS disease features [236,237]. 

Despite the many advantages and knowledge acquired by the use of these ALS mice, rats and guinea pigs, rodent modeling requires a high maintenance cost and a time-consuming investigation.

### 3.7. Canine ALS Modeling

Due to clinical and molecular similarities, canine degenerative myelopathy (CDM) is the only spontaneously occurring pathology that represents a model for human SOD1-related ALS [238]. Precisely, CDM is characterized by the progressive degeneration of axons, muscle atrophy, hyporeflexia, astrocytosis, peripheral demyelination and SOD1 inclusions [238,239,240]. Interestingly, T18S and E40K missense mutations in the canine *SOD1* gene are the only mutations reported as CDM causative [239,241]. Different from ALS, whose *SOD1* causative mutations are inherited as dominant, CDM shows recessive inheritance with a reduced penetrance [239]. Both T18S and E40K mutations do not cause disruption in the dismutase domain and impact only on protein hydrophobicity but not stability although promote the formation of aggregates and thus suggesting a gain-of-toxic-function [242,243].

Recent insights about the glia contribution to CDM describe a demyelination of oligodendrocytes [240], an increase of arginase 1-expressing microglia in the proximity of motor neurons [244] and the upregulation of CB_2_ receptors in activated astrocytes as the main cellular responses and biological markers of the disease [245]. Of note, these pathological features confirm those previously described in ALS rodent models and in patients [206,246,247].

Taking into account the high pathogenic similarity between spontaneous CDM and SOD1-ALS and the analogous complexity of human and canine nervous system, studies on dog tissues may help to better dissect the still unknown pathomechanisms of ALS and thus support the progress for therapeutic developments. However, it should be considered that dogs with CDM are often euthanized at early phases of the disease so the tissues potentially available for investigative studies can provide information only about early disease stages.

### 3.8. Non-Human Primate Models of ALS

Non-human primates (NHPs) are the evolutionarily closest species to humans and share many structural, cognitive and functional features. In particular, they have the capability to exert high brain functions such as thinking, reasoning, decision making and judging because of the development of a complex network of brain connections involving the neocortex and prefrontal cortex [248,249]. These features, together with neuroanatomical and genetic similarities, make NHPs a very desirable ALS model. 

*Callithrix jacchus*, the marmoset, is one of the most commonly used NHPs to validate potential therapeutic strategies and to generate neurodegenerative diseases models thanks to its small size, easy handling and rapidity to obtain transgenic forms [250,251,252]. For instance, a SOD1-ALS marmoset model was obtained through the intrathecal delivery of an adeno-associated virus encoding an artificial *SOD1*-specific microRNA, determining the reduction of SOD1 levels both in motor neurons and spinal cord sections [253]. By the same technique, a *Macaca fascicularis* SOD1-ALS model was recently obtained [254]. Likewise, a stereotaxic injection in *Callithrix jacchus* of a *FUS*-targeting shRNA was used to obtain a FUS-ALS marmoset model [255]. A TDP-43 overexpressing model was realized in *Macaca fascicularis* through the cervical injection of an adeno-associated virus containing a TDP-43 coding sequence. All of these monkeys exhibited muscle atrophy, progressive motor weakness, TDP-43 mislocalization and Cystatin C aggregates reminiscent of ALS patients [256], thus confirming the faithful correspondence between patient symptoms and what can be reproduced in monkeys.

Despite the clear advantage of these models, gene therapy approaches in NHPs need without a doubt further development but because of the ethical issue complexity, they are only a promising route to treat ALS. 

## 4. Concluding Remarks

In this review we have described how several unicellular and multicellular organisms including vertebrate and invertebrate eukaryotes such as yeast, worms, flies, zebrafish, mice, rats, guinea pigs, dogs and, more recently, non-human primates, can serve as model systems to efficiently untangle the complicated ALS pathogenesis (Table 1). Indeed, a thorough molecular analysis demonstrated that each of these organisms has greatly contributed to highlighting specific features of the disease among which are excitotoxicity, oxidative stress, endoplasmic reticulum stress, mitophagy, autophagy, proteinopathy, gliosis and inflammation. However, by analyzing all of the major available information, we have come to the conclusion that understanding the causes and mechanisms of ALS would better require the concurrent and comparative analysis of these animal models although harnessing these powerful organisms still poses numerous challenges about their translational power. Indeed, comparative research deals with models based on interspecies and intraspecies variations can sometimes even complicate the task of explaining the disease itself.

If we then ask why these models have become so widespread in preclinical analyses, we have to recognize their high flexibility in reproducing a few of the main clinical features of the disease, at least in part and in their own ways. By translationally investigating lifespan, motor capabilities (through swimming, climbing and running tests), morphological/histological modifications, motor neuron excitability and signal transduction mechanisms at cellular and molecular levels, these organisms have indeed proven to be instrumental in identifying new efficacious genetic modifiers and therapeutic agents against the insurgence and progression of the disease. Nevertheless, we are aware that they will never become surrogates for clinical testing.

Overall, this review has described common features but also discrepancies among experimental organisms in ALS, underlining new insights and emerging roles. In other words, we have illustrated the promises and problems of extrapolating ALS findings from animal models.

## Figures and Tables

**Table 1:** Where and why modeling amyotrophic lateral sclerosis (ALS).

Where	Why	Why Not
**Cell Lines**	Neuron-like phenotype	Lack of system complexity
**iPSCs**	fALS and sALS modeling	Lack of system complexity
**Yeast**	Very suitable for investigating cytotoxicity and proteinopathy aspects	Unicellular organism
**Worms**	Easy and precise investigation of neuronal circuits	Brain and blood absence
**Flies**	Fast and reliable reproduction of ALS symptoms	Anatomical difference with the human nervous system
**Zebrafish**	Nervous system and neurotransmitters similar to humans	Lack of upper motor neurons
**Rodents**	Nervous system and neurotransmitters very similar to humans	High maintenance costs and time-consuming investigation
**Canines**	Spontaneously occurring disease, nervous system as complex as humans	Only early-stage disease tissues available
**Non-Human Primates**	Neuroanatomical, cognitive and functional similarities with humans	Ethical issues

## Data Availability

Not applicable.
